# Supplementary material for: A Comparative Study of the Effectiveness of Pharmacopuncture Therapy for Chronic Neck Pain: A Pragmatic, Randomized, Controlled Trial
Source: J Clin Med. 2021 Dec 21;11(1):12. doi: 10.3390/jcm11010012 (PMC8745044; doi:10.3390/jcm11010012)
Supplement: Supplementary file 1 [file jcm-11-00012-s001.zip › jcm-1476610-supplementary.pdf]

Table S1. Schedule of the participants.

Time point (week)	Screening	Enrollment allocation	Active treatment after allocation				Follow-up		
	-1	0 (Baseline)	1	2	3	4	5	8	12
Visit window		**± 3**	**± 3**	**± 3**	**± 3**	**± 3**	**± 3**	**± 7**	**± 7**
**Enrollment**									
Eligibility screening	○	○	–	–	–	–	–	–	–
Written informed consent	○	–	–	–	–	–	–	–	–
Vital signs	○	○	○	○	○	○	○	○	○
Sociodemographic characteristics, medical history (e.g., neck pain, medication history)	○	–	–	–	–	–	–	–	–
Cervical spine X-ray	○	–	–	–	–	–	–	–	–
Randomized allocation	–	○	–	–	–	–	–	–	–
**Interventions**									
Treatment for the pharmacopuncture group (experimental group)			← 2 (1-3) times/week →						
Treatment for the PT group (control group)			← 2 (1-3) times/week →						
**Assessments**									
Symptoms and change	–	–	○	○	○	○	○	○	○
NRS scores for neck and arm pain	–	–	○	○	○	○	○	○	○
VAS score for neck pain	○	○	○	○	○	○	○	○	○
VAS score for arm pain	–	–	○	○	○	○	○	○	○
NPQ	–	–	○	–	–	–	○	○	○
NDI	–	–	○	–	–	–	○	○	○
PGIC	–	–	–	–	–	–	○	○	○
SF-12	–	–	○	–	–	–	○	○	○
EQ-5D-5L	–	–	○	–	–	–	○	○	○
Physical examination	–	–	○	–	–	–	○	–	–
Drug use	○	○	○	○	○	○	○	○	○
Evaluation of medical costs	–	–	○	–	–	–	○	○	○
Evaluation of time cost				○					
Evaluation of lost work productivity			○	○	○	○	○	○	○
Credibility and expectancy			○						
Blood analysis			○				○		
Adverse events		○	← Every visit →				○	○	○

PT, physical therapy; ***NRS***, numeric rating scale; ***VAS***, visual analogue scale; ***NPQ***, Northwick Park Questionnaire; ***NDI***, neck disability index; ***PGIC***, patient global impression of change, ***SF-12***, 12-Item Short Form Health Survey; ***EQ-5D-5L***, EuroQoL 5-dimension 5-level instrument.

Table S2. List of treatments provided to the patients.

Intervention	N (%)	Mean ± SD
**Pharmacopuncture group (n = 49)**		
Shinbaro 2	47 (95.9)	8.4 ± 3.0
Harpagophytum procumbens	17 (34.7)	7.2 ± 1.8
Shinbaro 1	2 (4.1)	8 ± 0
**Physical therapy group (n = 49)**		
Interferential current therapy	36 (73.5)	7.4 ± 1.7
Deep heat therapy	31 (63.3)	6.0 ± 3.2
Superficial heat therapy	13 (26.5)	7.9 ± 0.3
Laser therapy	13 (26.5)	6.4 ± 1
Transcutaneous electrical nerve stimulation	12 (24.5)	6.5 ± 2.2
Extracorporeal shock wave therapy	6 (12.2)	4.3 ± 2.4

The number (%) of patients in both groups who received each treatment are shown. Multiple uses were allowed. ***SD***, standard deviation.

Table S3. Primary and secondary outcomes according to the per-protocol analysis .

		Week 5	Week 8	Week 12
**VAS score for neck pain**	Pharmacopuncture (n = 47)	33.74 (28.49, 39.00)	34.08 (20.48, 47.68)	35 (15.12, 54.88)
	PT (n = 46)	17.85 (12.64, 23.06)	21.73 (1.79, 41.68)	27.28 (3.46, 51.10)
	Difference in decrease (95% CI)	16.60 (9.73, 23.47)	13.16 (1.35, 24.97)	8.40 (-1.97, 18.78)
	P-value	<.0001	0.029	0.112
**VAS score for arm pain**	Pharmacopuncture (n = 47)	18.19 (12.07, 24.32)	18.55 (10.29, 26.8)	21.10 (13.18, 29.02)
	PT (n = 46)	10.5 (4.31, 16.69)	10.83 (2.42, 19.23)	11.24 (1.92, 20.56)
	Difference in decrease (95% CI)	5.53 (-1.24, 12.30)	5.44 (-2.30, 13.18)	7.21 (-1.44, 15.86)
	P-value	0.108	0.168	0.102
**NRS score for neck pain**	Pharmacopuncture (n = 47)	3.28 (2.75, 3.8)	3.26 (2.65, 3.86)	3.40 (2.82, 3.99)
	PT (n = 46)	1.70 (1.16, 2.23)	1.83 (1.29, 2.36)	2.22 (1.60, 2.83)
	Difference in decrease (95% CI)	1.68 (1.01, 2.35)	1.51 (0.75, 2.27)	1.27 (0.48, 2.06)
	P-value	<.0001	<.0001	0.002
**NRS score for arm pain**	Pharmacopuncture (n = 47)	1.66 (1.05, 2.27)	1.72 (1.03, 2.41)	2.04 (1.37, 2.71)
	PT (n = 46)	1.15 (0.57, 1.73)	1.21 (0.65, 1.77)	1.13 (0.28, 1.99)
	Difference in decrease (95% CI)	0.40 (-0.23, 1.03)	0.41 (-0.31, 1.13)	0.78 (-0.07, 1.64)
	P-value	0.212	0.263	0.071
**NDI**	Pharmacopuncture (n = 47)	14.78 (11.39, 18.16)	17.12 (13.13, 21.11)	17.79 (13.63, 21.95)
	PT (n = 46)	7.73 (5.06, 10.40)	9.78 (6.86, 12.71)	10.31 (6.99, 13.63)

	Difference in decrease (95% CI)	5.35 (1.42, 9.27)	5.39 (0.88, 9.90)	5.50 (0.59, 10.41)
	P-value	0.008	0.019	0.029
**NPQ**	Pharmacopuncture (n = 47)	17.62 (14.08, 21.16)	17.91 (13.48, 22.33)	20.52 (16.37, 24.67)
	PT (n = 46)	9.77 (6.72, 12.82)	11.31 (8.09, 14.52)	11.93 (8.17, 15.68)
	Difference in decrease (95% CI	6.43 (2.16, 10.70)	4.89 (-0.08, 9.86)	7.04 (1.84, 12.25)
	P-value	0.004	0.054	0.009
**EQ-5D-5L**	Pharmacopuncture (n = 47)	-0.10 (-0.14, -0.06)	-0.11 (-0.15, -0.07)	-0.14 (-0.18, -0.09)
	PT (n = 46)	-0.04 (-0.07, -0.01)	-0.04 (-0.07, -0.01)	-0.06 (-0.1, -0.03)
	Difference in decrease (95% CI)	-0.01 (-0.05, 0.02)	-0.01 (-0.04, 0.02)	-0.02 (-0.06, 0.03)
	P-value	0.405	0.647	0.446
**SF-12 (MCS)**	Pharmacopuncture (n = 47)	-4.44 (-7.11, -1.77)	-4.21 (-7.57, -0.84)	-5.99 (-9.31, -2.66)
	PT (n = 46)	-4.41 (-7.34, -1.47)	-4.03 (-6.95, -1.12)	-4.72 (-7.6, -1.83)
	Difference in decrease (95% CI)	1.42 (-1.76, 4.59)	1.36 (-2.35, 5.06)	0.24 (-3.41, 3.90)
	P-value	0.379	0.473	0.895
**SF-12 (PCS)**	Pharmacopuncture (n = 47)	-5.53 (-8.03, -3.03)	-7.02 (-9.29, -4.76)	-7.23 (-9.62, -4.83)
	PT (n = 46)	-1.84 (-4.12, 0.44)	-2.81 (-5.14, -0.48)	-2.98 (-5.40, -0.55)
	Difference in decrease (95% CI)	-2.59 (-5.41, 0.24)	-3.10 (-5.76, -0.44)	-3.08 (-5.84, -0.32)
	P-value	0.072	0.023	0.029
**PGIC**	Pharmacopuncture (n = 47)	2.36 (2.17, 2.55)	2.51 (2.25, 2.77)	2.34 (2.11, 2.57)
	PT (n = 46)	3.11 (2.90, 3.32)	3.16 (-3.04, 9.36)	3.17 (2.93, 3.42)
	Difference in decrease (95% CI)	-0.75 (-1.02, -0.47)	-0.65 (-1.03, -0.27)	-0.83 (-1.17, -0.5)
	P-value	<.0001	0.001	<.0001

Effectiveness outcomes were assessed as the decrease from the baseline levels. Between-group differences were analyzed through an analysis of covariance with adjustment for the baseline values except patient global impression of change. The primary endpoint was week 5. Missing values were added through multiple imputation. Estimates for each group and between-group differences in the decrease at each time point are displayed with the 95% confidence intervals (CIs). ***PT***, physical therapy; ***CI***, confidence interval; ***VAS***, visual analogue scale; ***NRS***, numeric rating scale; ***NDI,*** neck disability index; ***NPQ***, Northwick Park Questionnaire; ***SF-12***, 12-Item Short Form Health Survey; ***EQ-5D-5L***, EuroQoL 5-dimension 5-level instrument; ***MCS***, mental component summary; ***PCS,*** physical component summary; ***PGIC***, patient global impression of change. The VAS, NRS, EQ-5D-5L, SF-12, and PGIC were measured as scores. The NDI and NPQ were calculated as percentages. The PGIC was assessed using a scale from 1 (improved) to 7 (worsened), with a lower score indicating more improvement. Between-group differences were analyzed by performing an independent t-test of the endpoint values.

Table S4. Analysis of between-group differences with the linear mixed model.

	Week 2	Week 3	Week 4	Week 5	Week 8	Week 12
VAS score for neck pain						
Pharmacopuncture*	8.64 (5.30, 11.98)	17.38 (12.87, 21.89)	27.04 (22.00, 32.08)	33.73 (28.59, 38.87)	34.51 (27.27, 41.75)	35.44 (29.25, 41.64)
PT*	5.04 (2.85, 7.24)	8.79 (6.04, 11.54)	12.08 (8.50, 15.67)	17.10 (12.01, 22.20)	19.80 (13.19, 26.41)	24.48 (16.76, 32.21)
Group*visit	3.45 (-0.5, 7.41)	8.32 (3.14, 13.5)	14.5 (8.45, 20.55)	16.33 (9.2, 23.46)	15.49 (6.88, 24.11)	11.23 (2.92, 19.55)
P-value	0.0864	0.0019	<.0001	<.0001	0.0006	0.0086
VAS score for arm pain						
Pharmacopuncture*	2.74 (-1.94, 7.43)	9.64 (4.73, 14.55)	13.47 (8.65, 18.29)	18.65 (12.58, 24.71)	18.80 (11.07, 26.54)	22.44 (14.65, 30.24)
PT*	2.85 (-1.42, 7.11)	3.36 (-0.85, 7.57)	8.52 (3.90, 13.15)	10.42 (4.45, 16.38)	13.77 (7.92, 19.63)	15.55 (6.41, 24.68)
Group*visit	0.18 (-5.99, 6.35)	6.33 (0.06, 12.59)	5.06 (-1.43, 11.55)	8.47 (0.14, 16.8)	9.82 (0.59, 19.06)	8.97 (-1.19, 19.12)
P-value	0.9543	0.0477	0.1251	0.0464	0.0373	0.0828
NRS score for neck pain						
Pharmacopuncture*	0.74 (0.40, 1.09)	1.55 (1.09, 2.02)	2.66 (2.16, 3.16)	3.29 (2.78, 3.81)	3.29 (2.70, 3.88)	3.42 (2.85, 3.99)
PT*	0.57 (0.29, 0.84)	0.87 (0.58, 1.16)	1.29 (0.90, 1.68)	1.67 (1.15, 2.18)	1.81 (1.30, 2.32)	2.19 (1.59, 2.78)
Group*visit	0.17 (-0.26, 0.6)	0.68 (0.14, 1.22)	1.36 (0.74, 1.97)	1.60 (0.88, 2.32)	1.48 (0.72, 2.24)	1.21 (0.39, 2.02)
P-value	0.4325	0.0135	<.0001	<.0001	0.0002	0.004
NRS of arm pain						
Pharmacopuncture*	0.13 (-0.31, 0.57)	0.74 (0.30, 1.19)	1.17 (0.70, 1.64)	1.69 (1.09, 2.28)	1.77 (1.09, 2.45)	2.08 (1.42, 2.74)
PT*	0.35 (-0.09, 0.79)	0.47 (0.08, 0.86)	1.04 (0.59, 1.49)	1.17 (0.61, 1.72)	1.38 (0.86, 1.91)	1.19 (0.36, 2.01)
Group*visit	-0.22 (-0.82, 0.38)	0.31 (-0.27, 0.88)	0.16 (-0.47, 0.8)	0.55 (-0.25, 1.35)	0.60 (-0.32, 1.51)	0.93 (-0.11, 1.96)
P-value	0.4692	0.2924	0.6132	0.1776	0.1983	0.0778
NDI						
Pharmacopuncture*				14.88 (11.56, 18.20)	17.18 (13.27, 21.09)	17.75 (13.68, 21.83)
PT*				7.95 (5.34, 10.56)	10.07 (7.45, 12.69)	10.55 (7.34, 13.75)
Group*visit				6.72 (2.56, 10.88)	6.94 (2.33, 11.56)	6.96 (1.85, 12.06)

P-value	0.0018	0.0036	0.0081
NPQ			
Pharmacopuncture*	17.66 (14.19, 21.12)	18.11 (13.76, 22.46)	20.49 (16.43, 24.55)
PT*	9.88 (6.93, 12.84)	11.47 (8.45, 14.50)	12.18 (8.56, 15.80)
Group*visit	7.45 (2.95, 11.95)	6.43 (1.2, 11.66)	7.96 (2.58, 13.34)
P-value	0.0014	0.0165	0.0041
EQ-5D-5L			
Pharmacopuncture*	-0.10 (-0.13, -0.06)	-0.11 (-0.15, -0.07)	-0.13 (-0.18, -0.09)
PT*	-0.04 (-0.06, -0.01)	-0.04 (-0.08, -0.01)	-0.06 (-0.10, -0.03)
Group*visit	-0.06 (-0.11, -0.02)	-0.07 (-0.12, -0.02)	-0.07 (-0.12, -0.02)
P-value	0.0053	0.0075	0.011
SF-12 (MCS)			
Pharmacopuncture*	-4.36 (-6.98, -1.74)	-4.09 (-7.39, -0.79)	-5.74 (-9.03, -2.46)
PT*	-4.45 (-7.28, -1.62)	-4.50 (-7.43, -1.56)	-4.98 (-7.77, -2.19)
Group*visit	0.07 (-3.71, 3.86)	0.33 (-3.99, 4.64)	-0.78 (-5.01, 3.45)
P-value	0.9699	0.8803	0.7153
SF-12 (PCS)			
Pharmacopuncture*	-5.58 (-8.03, -3.13)	-7.00 (-9.22, -4.79)	-7.30 (-9.65, -4.96)
PT*	-1.72 (-3.92, 0.48)	-2.59 (-4.82, -0.36)	-2.82 (-5.15, -0.49)
Group*visit	-3.73 (-6.97, -0.5)	-4.39 (-7.46, -1.31)	-4.34 (-7.6, -1.09)
P-value	0.0242	0.0056	0.0094

The estimates for each group and between-group differences in the pain decrease at each time point are displayed with the 95% confidence intervals (CIs). ***PT***, physical therapy; ***VAS***, visual analogue scale; ***NRS***, numeric rating scale; ***NDI,*** neck disability index; ***NPQ***, Northwick Park Questionnaire; ***SF-12***, 12-Item Short Form Health Survey; ***EQ-5D-5L***, EuroQoL 5 dimension 5-level instrument; ***MCS***, mental component summary; ***PCS,*** physical component summary; ***PGIC***, patient global impression of change.

Table S5. Summary of treatment-related adverse events.

	**Pharmacopuncture**	**Physical therapy**
Treatment-related adverse events	2	0
Gastrointestinal disorders	0	0
Musculoskeletal and connective tissue disorders	0	0
Nervous system disorders	0	0
Symptom-related skin disorders	2	0
Serious adverse events	0	0
Severity		
Mild	2	0
Moderate	0	0
Severe	0	0
